# Supplementary material for: Screening of Bifidobacteria with Probiotic Potential from Healthy Infant Feces by Using 2′-Fucosyllactose
Source: Foods. 2023 Feb 17;12(4):858. doi: 10.3390/foods12040858 (PMC9957139; doi:10.3390/foods12040858)
Supplement: Supplementary file 1 [file foods-12-00858-s001.zip › foods-2159396-supplementary.pdf]

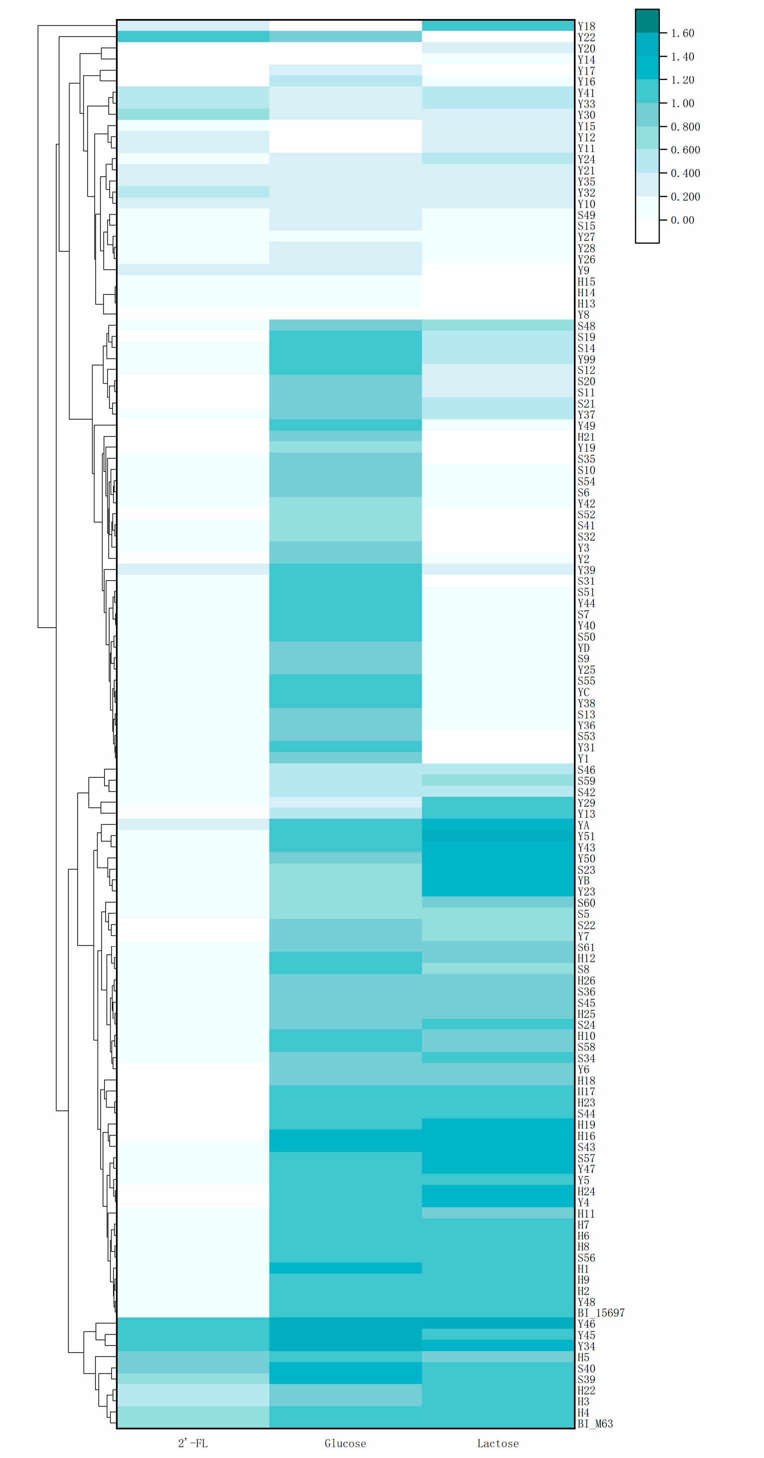

Figure S1: Maximum bacterial growth (change in optical density at 595 nm) of 126 isolates in the presence of 1% glucose, lactose, and 2′-fucosyllactose (2′-FL) during 48 h of incubation.

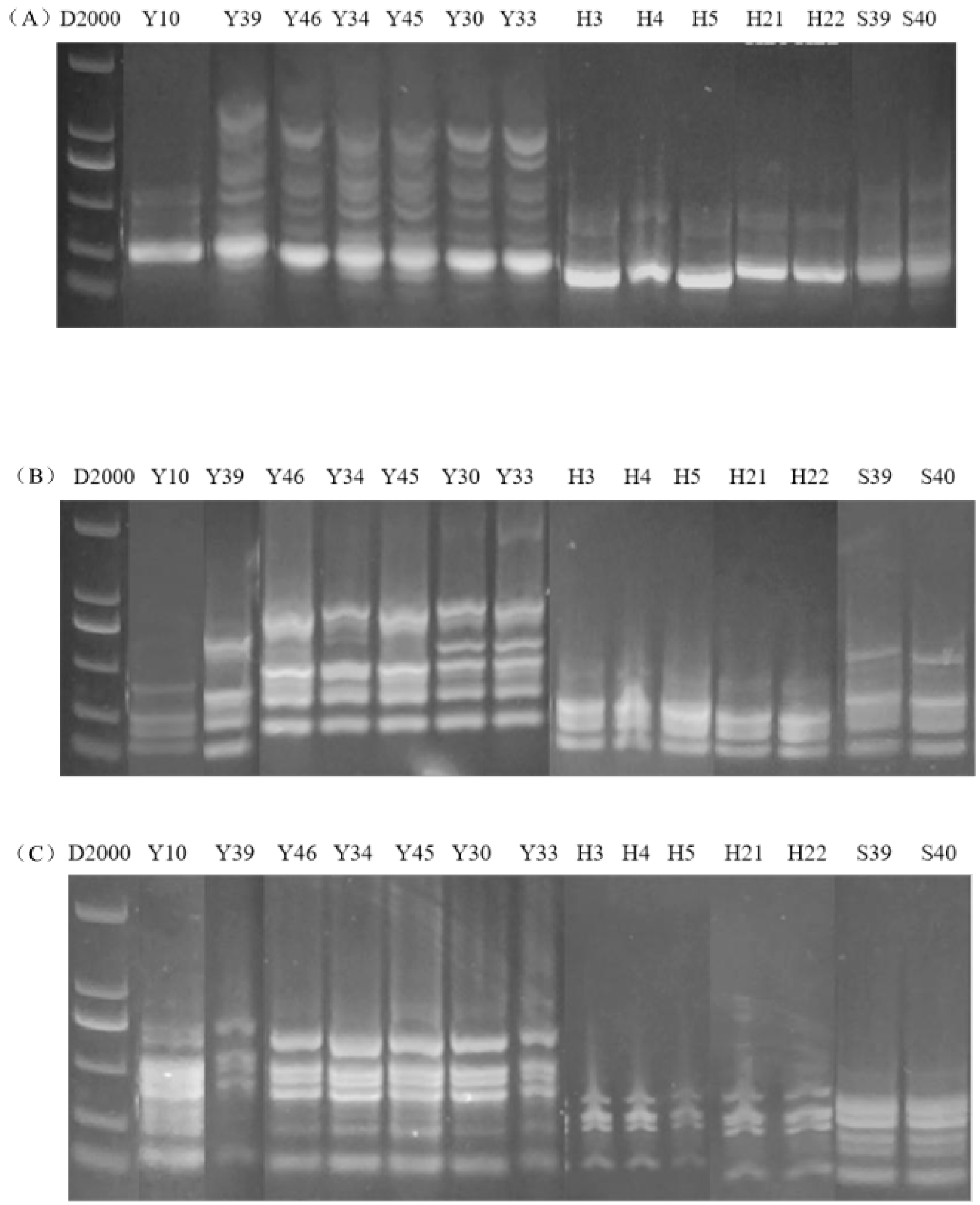

Figure S2 RAPD electropherogram of isolated bifidobacteria able to use 2′-fucosyllactose effectively using three random primers S23 (A), S228 (B), and S396 (C)
